# Asymmetric expression of proteins in the granules of the placentomal Binucleate cells in *Giraffa camelopardalis*^†^

**DOI:** 10.1093/biolre/ioab247

**Published:** 2022-01-17

**Authors:** F B P Wooding, A J Forhead, S Wilsher, W R Allen, R M Roberts, J A Green, J F Beckers, N Melo Sousa, G Charpigny

**Affiliations:** The Physiological Laboratory, University of Cambridge, Downing Site, Cambridge, CB2 3EG; The Physiological Laboratory, University of Cambridge, Downing Site, Cambridge, CB2 3EG; The Paul Mellon Laboratory of Equine Reproduction, Newmarket, Suffolk, CB8 9BJ; The Paul Mellon Laboratory of Equine Reproduction, Newmarket, Suffolk, CB8 9BJ; Division of Animal Sciences, University of Missouri, Columbia, Missouri, USA; Division of Animal Sciences, University of Missouri, Columbia, Missouri, USA; Physiologie de la Reproduction, Faculte de Medecine Veterinaire, B-4000, Liege, Belgique; Physiologie de la Reproduction, Faculte de Medecine Veterinaire, B-4000, Liege, Belgique; INRA, UMR1198, Biologie Devel Reprod, F-78532 Jouy et Josas, France

**Keywords:** Ruminant placenta, Trophoblast binucleate cell, Giraffe granule protein asymmetry

## Abstract

Mature granulated trophoblast binucleate cells (BNC) have been found in all ruminant placentas examined histologically so far. BNC are normally fairly evenly distributed throughout the fetal villus and all their granules contain a similar variety of hormones and pregnancy associated glycoproteins (PAGs). Only the Giraffe is reported to show a different BNC protein expression, this paper is designed to investigate that. Gold labelled Lectin histochemistry and protein immunocytochemistry were used on deplasticised 1 μm sections of a wide variety of ruminant placentomes with a wide range of antibodies and lectins. In the Giraffe placentomes, even though the lectin histochemistry shows an even distribution of BNC throughout the trophoblast of the placental villi, the protein expression in the BNC granules is limited to the BNC either in the apex or the base of the villi. Placental lactogens and Prolactin (PRL) are present only in basally situated BNC: PAGs only in the apical BNC. PRL is only found in the Giraffe BNC which react with many fewer of the wide range of antibodies used here to investigate the uniformity of protein expression in ruminant BNC. The possible relevance of these differences to ruminant function and evolution is considered to provide a further example of the versatility of the BNC system.

## Introduction

Mature granulated trophoblast BNC have been found in all ruminant placentas examined histologically so far [1–3]. Recent investigations suggest that all mature BNC undergo the same characteristic migration out of the trophoblast followed by fusion with a uterine epithelial cell or derivative to form fetomaternal tissue throughout pregnancy [4]. The granules are released to the maternal compartment by exocytosis from this fetomaternal tissue. BNC are normally fairly evenly distributed throughout the fetal villus and all their granules contain a similar variety of hormones and pregnancy associated glycoproteins (PAGs) [1].

The one exception to this uniformity so far is the giraffe, (*Giraffa camelopardalis*) which is reported to show BNC whose granules react uniquely with a Prolactin antibody but are restricted to the base of the fetal villi [2].

The giraffoid clade now consists only of Giraffes, Okapis and Pronghorns [5] but they were much more widespread in the Miocene era [6]. They are now restricted to a few species in marked comparison with the enormous expansion of the other ruminant clades, in particular the Bovidae. Fortunately samples of Okapi and Pronghorn placentas were available, as well as a wide variety of other ruminant placentas [Supplementary Table S1].

Antibodies to Prolactin and purified Placental lactogens from three different species were used (Table 1) together with examples from the two phylogenetically distinct ancient and modern groups of PAGs [7, 14].

**Table 1:** Antibodies and Lectins used

Abbreviations	Antibody	Antibody origins	
“ancient”PAGs			
RA	Anti native bovine-PAGs	Wooding et al 2005 [13]	
RD	Anti-boPAG 2		
BJ	Anti-boPAG 2	Beckers et al 1994 [18]	R438
“new”PAGs			
RB	Anti native bo-PAGs	Wooding et al 2005 [13]	
RC	Anti –ovinePAG 1		
RU			
FPIR	Anti bo PAG 1	Touzard et al 2013 [14]	
BG	Anti-bo PAG 1	Zoli et al 1991 [15]	R726
Buffalo PAGs			
BK	Anti-wbPAGs	Barbato et al 2013 [19]	R858
BM	Anti-wbPAGs (different N terminal sequence)		R859
Goat PAGs			
BB	Anti-capPAG55 + 59 kDa	Garbayo et al 1998 [16]	R708
BE	Anti-capPAG55 + 62 kDa		R706
Ovine PAG	Anti-ovPAG57	El-Amiri et al 2004 [20]	R780
BN	Anti-bovine PAG 1	Zoli et al 1991 [15]	
Placental Lactogens			
Friesen oPL	Anti ovinePL	Chan et al 1978 [22]	
BF	Anti- bovine PL	Alvarez-Oxiley et al 2007 [17]	
PROLACTIN	Anti humanPRL	NIH	
SBU3	Monoclonal ab, Homologous to ovine PAG	Gogolin-Ewens et al,1987 [21]	
LECTINS	Vector Laboratories,	Peterboro, UK	
DSA			
DBA			
ePHA			
lPHA			

^*^For the differences between “ancient” and “new” PAGs see [14], the Touzard et al reference and Wallace et al [7].

This paper reports a qualitative and quantitative study of ruminant BNC position and granule content in the species detailed above using the range of antibodies and lectins.

This variety of results should facilitate investigation of the possible relevance of any differences to ruminant function and evolution.

## Methods and materials

### Animals

Mid to late pregnant placental material was used from a wide variety of ruminants collected over many years (Supplemental Table S1 and see Wooding references in [1]). They were fixed by aldehyde immersion or perfusion.

Wild animals were shot as part of Wild life management or culling procedures and placentomes removed and immersion fixed within 20 minutes of the death of the animals. Fixatives used included Bouins, phosphate buffered Paraformaldehyde or Glutaraldehyde, and Surgipath (methyl alcohol and formaldehyde). The quality of fixation varied, but crucially, all produced comparable results with the antibodies and lectins (Table 1) used on the placentome sections.

Small pieces of Horse Anterior Pituitary were also fixed and processed as the placentome samples.

For details of the animals and origins see Supplemental Table S1.

At least two placentomes from a single animal were used in the case of the Wildebeest, at least two or more animals from each of the other species were used.

A central slice was cut from each fixed placentome. “Matchstick” samples from the central region of each slice from maternal to fetal edge were used. The samples were then embedded in epoxy resin with no osmium postfixation.

Semithin sections were cut, picked up on cover glass squares treated with APES, deresinated in sodium ethoxide and thoroughly washed in PBS.

### Immunocytochemistry

The cover glass squares were then floated section side down on drops of antibody (see Table 1) followed by immunogold colloid (goat anti-rabbit G5, Jackson Immunoresearch Labs, USA) and then intensified with silver reagent (Aurion, Wagenigen, Netherlands). The coverslips were washed thoroughly between each incubation.

The antibodies used (Table 1) were to purified lactogens or Pregnancy Associated Glycoproteins (PAGs) from a variety of species (see Supplementary Table S1) used at a dilution of 1:1000.

### Lectin histochemistry

The cover glass squares were floated section side down on drops of biotinylated lectins (see Table 1) followed by immunogold colloid.

(Goat antibiotin G5) and then intensified with silver reagent (Aurion, Wagenigen, Netherlands). The lectins used were chosen as the most BNC reactive although the silver enhancement did produce some background with the giraffe specimens.

The antibodies and lectins identified BNC granules in all species used. Controls with buffer substituted for antibody or lectin showed no significant labelling.

### Assessment of Labeling

Visual estimate of antibody reaction on BNC granules, scale used: —, no reactivity, (+) sporadic/occasional: +, ++, +++ indicate increasing levels of reactivity.

### Quantitation of BNC

The number of BNC visible in the X10 objective field of view was counted at random positions along the axis of the fetomaternal “matchstick” from the fetal to maternal side.

The area of the field of view was converted to mm ^2^ and the position along the “matchstick” estimated by eye.

The results for any particular lectin or antibody are consistent but the standard deviations are high mainly because of the variable areas of core villus connective tissue on the sections. To minimise this problem semiserial sections were used for any one comparative run.

## Results

**Figure 1:**
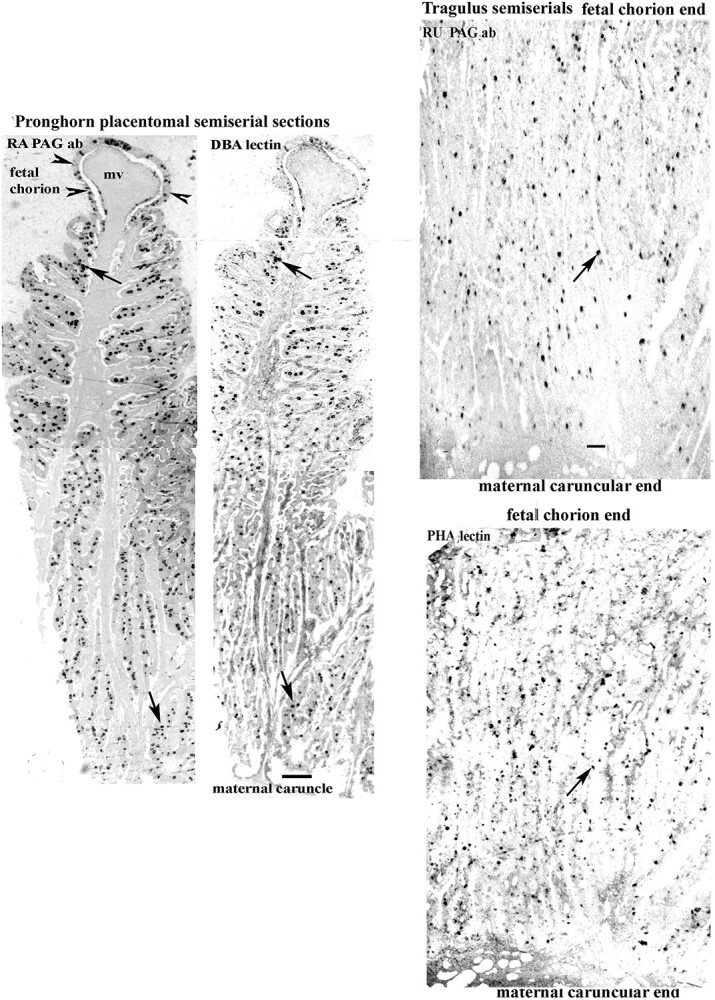
Pronghorn and Tragulus; placentomal semiserial sections showing similar localisations and even distributions of BNCs throughout the sections using PAG antibody or lectin localisations. Maternal villus, mv. Arrows indicate different levels of the same BNCs on the pairs of sections. Arrowheads identify the fetal basal chorionic layer on the Pronghorn. Scale bar = 250 μm. Tragulus; Scale bar = 80 μm.

Our results show a remarkable degree of uniformity in the binucleate cell granule immunoreactivity in thirteen of the fourteen ruminant species we examined (Tables 2 and 4, Supplementary Fig. S1, Fig. 1).

**Table 2:** Assessment of the immunoreactivity of non giraffoid Ruminant BNC

ANTIBODY	COW	EWE	BISON	Tragulus	Springbok	Impala	Red deer	Chinese water deer	Wildebeest	White tail	Wapiti
“ancient”PAGs											
RA	+++	+++	++	+++	+++		+	+++	++	+++	+++
RD	+++	+++	+	+++	++				++		+++
BJ	++		++					+	++	+	+++
“new”PAGs											
RB	+++	+++	+++	+++	++		+++	+	+++	+++	+++
RC	+	+++	+++	+++	++	++	+		+++	+++	+++
RU	++	+++	+++	++		++	+++	+++	+++	+++	+++
FPIR	+++	++		+++		++	++			+++	
BC	++	++	++	+++	++	+			+	+++	+++
BO	++	+++	++	+++	+++	+	++	++			
Buffalo PAGs											
BK	++	++	+++	+++	+	+	++	++	++	+++	+++
BM	++	+++	+++	+++			++	+++	+++	+++	
Goat PAGs											
BB	+++	+++	+++	+++	+++	+	+++	++	+++	+++	+++
BE	+++	+++	+++	++	+++	+		++	+++	+++	+++
Ovine PAG											
BN	+++	+++	+++	+++	+++	+	++	+	+++	+++	
SBU3	++	++		++	++	+			++	++	++
Placental Lactogens											
Friesen bPL	+++	++	+++	−		++	++	++	++	++	
BF	++	++	+++	−	−	+	++	+	+++	+	+++
PROLACTIN	−	−	−	−	−	−	−	−	−	−	−
SBU3	++	++		++	++	+			++	++	++
LECTINS											
DSA	++	++		+++						++	
DBA	+++			+++	+++	+++				+++	+++
ePHA				+++							
lPHA				+++							

Scale used: —, no reactivity, +, ++, +++ indicate increasing levels of reactivity. Empty oblong: assessment not done

We used a wide range of Pregnancy Associated Glycoproteins (PAGs) and Placental Lactogen antibodies (Table 1) raised against proteins purified from ruminant placentas of different species. The BNC granules were detected by gold labelled immunocytochemistry by most of the antibodies and lectins used on deplasticised araldite sections which included the full depth of the placentomal villi from maternal to fetal sides.

In the thirteen, BNC were evenly distributed throughout the fetal villi with no consistent indication of any zonation (Tables 2 and 4, Supplementary Fig. S1, Fig. 1).

There have been reports of differences in zonation [eg, 3], but there are no previous quantitative studies confirming them.

**Figure 2:**
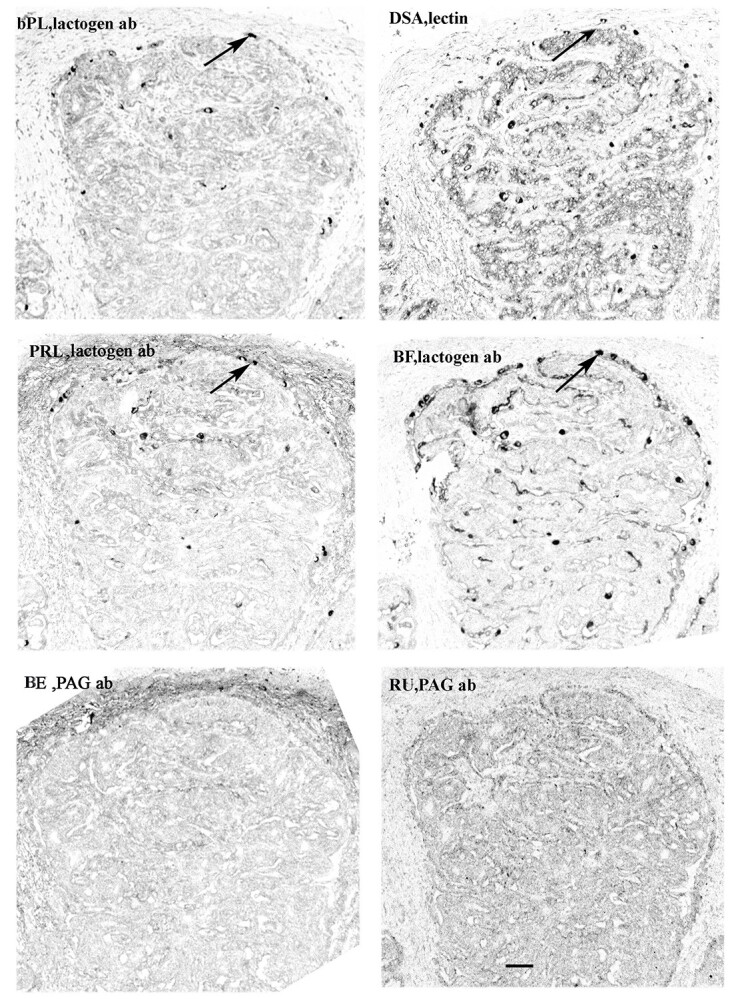
Giraffe placentomal fetal villus chorionic ends. Lactogen antibodies are present at the bases of the fetal chorionic villi (eg, at arrows); no BNCs can be identified using PAG antibody; BNCs are localised throughout the villi the using lectin. Arrows indicate different levels of probably the same BNCs on the sections using the lactogen antibody or lectin localisation. Scale bar =50 μm.

**Figure 3:**
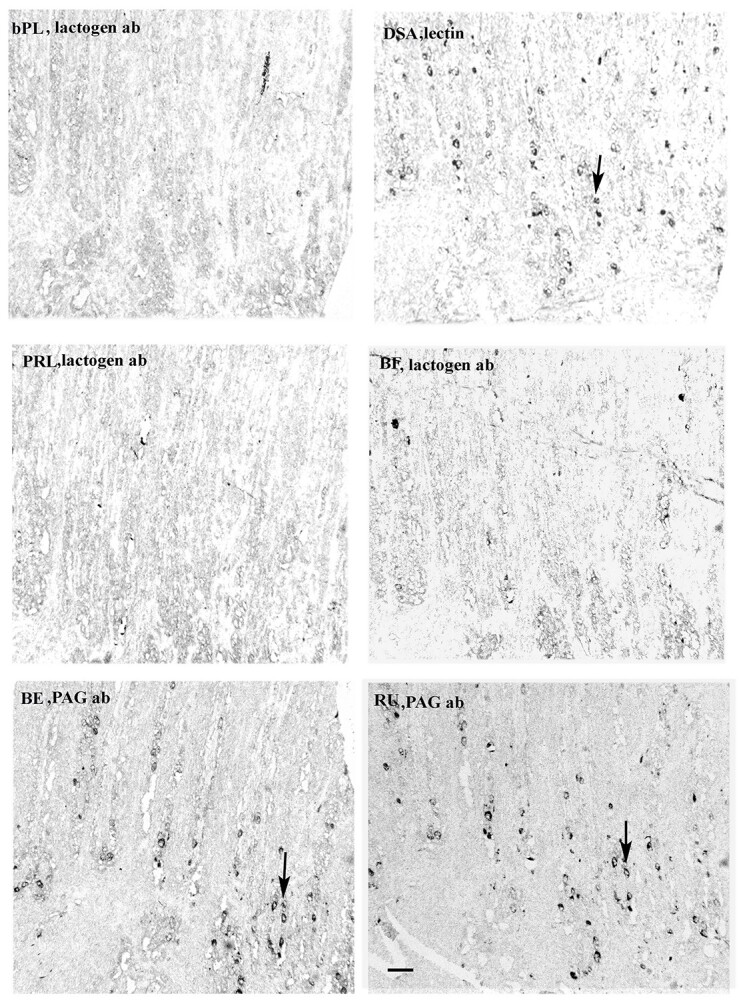
Giraffe placentomal fetal villus caruncular ends. PAG antibodies are present at the apices of the fetal chorionic villi (eg, at arrows); no BNCs can be identified using lactogen antibody; BNCs are localised throughout the villi using lectin. Arrows indicate BNCs in very similar positions using the PAG antibody or lectin localisation. Scale bar =50 μm.

All the BNC granules on any one section showed similar levels of expression of PAGs or lactogens or lectin reactivity, although the levels of expression differed between the various species and the PAGs, lactogens and lectins (Tables 3 and 5, Figs 1, 2, 3).

**Table 3:** The immunoreactivity of the BNC in the Giraffoid clade

	GIRAFFE			OKAPI	PRONGHORN		
ANTIBODY	Different Placentomes		Different animal		Different Placentomes		Different animal
“ancient”PAGs	G1A	G1B	G2		P1	P1G	P6
RA	(+)	(+)	(+)	+++	++++	++	++
RD	−	−	−	+++	+++	++	+++
BJ	−	+Mend	+Mend	++	++	+++	++
“new”PAGs							
RB	−	−	−	+	+	++	++
RC	−	+Mend	−	+	++	++	++
RU	++Mend only	+++M end	++Mend	+	+	+	+
FPIR	−	−	−	+	++	++	++
BC	−	−	−	−	+++	++	++
BO	−	−	−		++	+	+++
Buffalo PAGs	−						
BK	−	+Mend	−	+	++	++	++
BM	−	−	−	−	−	−	−
Goat PAGs							
BB	++Mend	++Mend	−	+++	++	++	++
BE	++Mend	++Mend	+++Mend	++	+++	+++	++
Ovine PAG							
BN	++Mend	++Mend	++Mend	−	++	+	+++
SBU3	−	−	−	−	−	−	−
Placental Lactogens							
Friesen bPL	++Fend	++Fend	+++Fend	−	−	−	−
BF	++Fend	++Fend	++Fend	+	−	−	−
PROLACTIN	++Fend	++Fend	++Fend	−	−	−	−
LECTINS							
DSA	+++		+++	+++	++	+++	+++
DBA	+		+		+	+	+
ePHA	++		+		++	+++	+++
lPHA	++				++	++	++

Scale used: —, no reactivity, (+) sporadic/occasional: +, ++, +++ indicate increasing levels of reactivity. Empty oblong: assessment not done.

M/F ends: Maternal or Fetal ends of the Fetal villus

In sharp contrast to the uniform immunoreactive BNC distribution in the 13 species described above, the Giraffe immunoreactive BNC distribution is unique. In this species the placental BNC granules react with many fewer of the antibodies used and when they do react it is in strictly localised areas, either the top or bottom 25% of the fetal villus length (Tables 3 and 5). This is more difficult to recognise when the entire length of the Giraffe sections are shown on Supplemental Fig. S2 and better appreciated at higher magnification on Figs. 2, 3).

All the antibodies expressed in the top 25% are against PAGs whereas all the basal group are anti PLs or Prolactin. The prolactin expression is unique to Giraffe, and it seems definite as the antibody can be absorbed against NIH prolactin on both placentome and Horse anterior pituitary sections (Supplementary Fig. S3).

One of the PLs (BF) does show a wider distribution than the top 25% of the villus but with a rapidly decreasing frequency towards the maternal end.

However localising giraffe BNC with the most strongly reacting lectin clearly demonstrates a uniform distribution throughout the fetal villi (Tables 3 and 4, Figs. 2, 3).

**Table 4:** Quantitation of the number of immunoreactive BNC per mm^2^ in four non giraffoid Ruminants

		VILLUS COUNT AREA	
SPECIES	ANTIBODY OR *LECTIN*	FETAL END 0 -50% n = 5	MATERNAL END 50 – 100% n = 5
COW(*Bos taurus*)	BB	80 ± 12	85 ± 11
	BC	90 ± 6	95 ± 9
	BF	90 ± 12	85 ± 10
	BG	88 ± 10	85 ± 15
	BK	88 ± 15	80 ± 10
	***DBA***	***45 ± 8***	***50 ± 11***
			
EWE (*Ovis aries*)	RA	68 ± 9	65 ± 10
	RB	36 ± 8	40 ± 11
	ovinePL	28 ± 10	30 ± 8
	bovinePL	30 ± 8	32 ± 10
			
TRAGULUS spp	RU	48 ± 14	50 ± 10
	***PHA***	***61 ± 9***	***69 ± 10***
			
PRONGHORN (*Antilocapra americana*)		97 ± 12	84 ± 15
	RU	78 ± 11	69 ± 12
	***DSA***	***68 ± 7***	***70 ± 7***

No significant differences were detected between fetal and maternal end counts

**Table 5:** Quantitative estimates of the number of immunoreactive BNC cells per mm^2^ in Giraffe

	Antibody or *Lectin*	Villus count position			
		0 – 25%	25% -50%	50% -75%	75%- 100%
Animal		Fetal end		Maternal end	
Giraffe A	***DSA***	61 ± 13	60 ± 11	63 ± 12	62 ± 14
	bovinePL	27 ± 6	1 ± 2	1 ± 1	0
	PRL	29 ± 6	1 ± 1	1 ± 1	0
	BF	67 ± 2	27 ± 8	11 ± 2	5 ± 2
	BE	0	0	0	65 ± 16
	RU	0	0	0	60 ± 9
Giraffe B	***DSA***	56 ± 9	58 ± 11	55 ± 11	56 ± 12
	bovinePL	28 ± 1	3 ± 1	2 ± 1	1 ± 1
	ovinePL	14 ± 2	0	0	0
	PRL	24 ± 1	0	0	0
	BF	53 ± 4	29 ± 9	24 ± 9	21 ± 6
	BE	0	0	0	26 ± 6
	RU	0	0	0	27 ± 5
	BB	0	0	0	20 ± 5
	BJ	0	0	0	14 ± 7
	BN	0	0	0	15 ± 5

No significant differences were detected between fetal and maternal end counts of the Lectins

The two other members of the giraffoid clade, Okapi (Okapi johnstoni) and Pronghorn (*Antilocapra americana*) show no zonation of immunoreactive BNC positioning and their BNC react with many more of the antibodies than the giraffe. (Table 3) They also show individual differences compared to the giraffe – no Prolactin or placental lactogen expression for example.

## Discussion

This paper establishes the asymmetry of the PAG and the PRL/lactogen distributions in the BNC granule content of the Giraffe placentomal villi compared with the uniformity of BNC granule content in the placentomal villi of all the other ruminant groups investigated so far. This asymmetry is also in contrast to the uniform distribution of the BNC in the Giraffe placentomal villi as clearly indicated by the lectin content of the BNC granules.

The Giraffe BNC granules react with many fewer of the antibodies used here but do show a unique content of PRL. This does not seem to be an artefact as shown by the ability to absorb the reactivity with pure PRL in both the giraffe placentome and the horse anterior pituitary sections (Supplementary Fig. S3).

There is also a clear difference in type at the two ends of the fetal villi with PRL and placental lactogens at the base and a range of PAGs at the top.

These peculiarities are not shared by the other members of the giraffoid clade, Okapi and Pronghorn, both of which show similar distribution of BNC granule immunoreactivity to the ruminant majority, although there are individual differences such as no reaction with the SBU3 or one of the Buffalo PAG antibodies.

It is perhaps of interest that the only other placental PRL trophoblast localisation reported is in the Elephant (*Loxodonta africana*) but the localisation is throughout the epithelium [8] and not restricted to any specialised cells.

The lectins used in this investigation were chosen for maximum reactivity. There are differences in lectin reactivity between Giraffoids and the other ruminants which are detailed in our previous papers [9–11].

The typical uniform BNC distribution with the unique formation of fetomaternal tissue by migration and fusion was established early in evolution as exemplified by the Tragulid line [12], and probably was a key factor in the success of the ruminant grouping. All of the mature ruminant placentas so far investigated show this characteristic pattern as do the BNC of Giraffe and Pronghorn [4].

There is no obvious or apparent functional reason for the PAG asymmetry unless it is necessary to establish a high concentration of PAGs in the caruncle. This could camouflage the area from invasive maternal lymphocytes to maintain the fetomaternal balance. This strategy has been suggested to be the case in the development of the bovine placental villi but no BNC asymmetry was necessary to produce that [13].

PRL and lactogens are considered to play important roles in the fetal metabolism but there is no reason to think that the asymmetric localisation would speed up the delivery to the fetus.

We have shown previously [2] that the stimulation of the protein expression is very precisely localised since the villus base (arcade) chorion presumably stimulates expression of BNC PRL and the lactogens but no significant PAGs whereas BNC in the adjacent intercotyledonary chorion express PAGs but no PRL or lactogens.

Both giraffe placentomal samples show a very similar unique BNC distribution and protein expression so it is unlikely to be a methodological artefact. Also the even distribution of the lectins here (and other antibodies used previously [2]) throughout the giraffe sections support this assumption.

Maybe it shows the versatility of the BNC system in solving the problems of maternofetal immunological balance in the extreme evolutionary niche occupied by the giraffe.

## Supplementary Material

FIG_S1_ioab247

FIG_S2_ioab247

FIG_S3_ioab247

Table_S1_ioab247

## Data Availability

The data underlying this article are available in the article and in its online supplementary material.
