# Supplementary figures and images for: Asymmetric expression of proteins in the granules of the placentomal Binucleate cells in Giraffa camelopardalis
Source: Biol Reprod. 2022 Jan 17;106(4):814–22. doi: 10.1093/biolre/ioab247 (PMC9305501; doi:10.1093/biolre/ioab247)

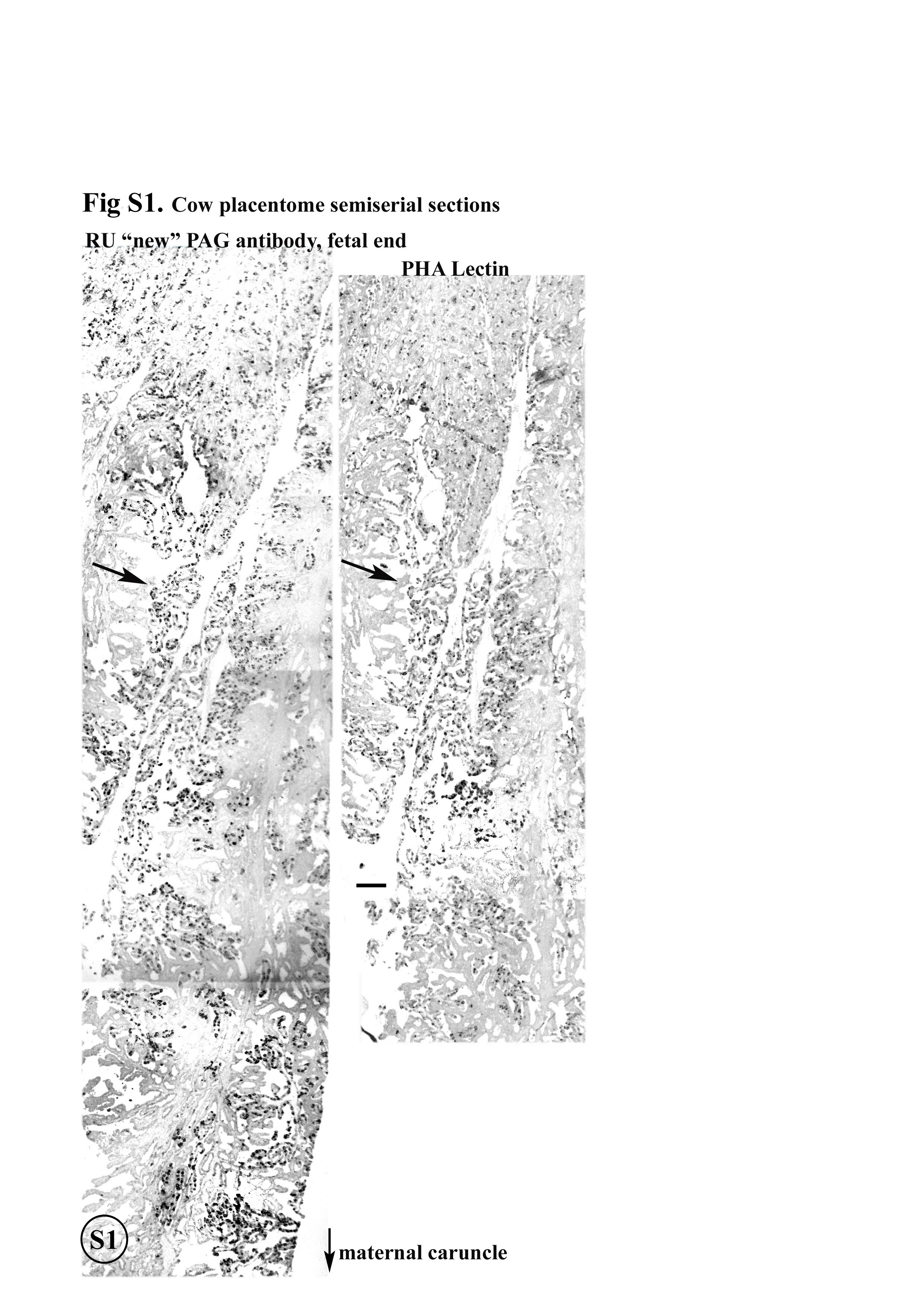

Supplement: FIG_S1_ioab247 [file fig_s1_ioab247.jpeg]

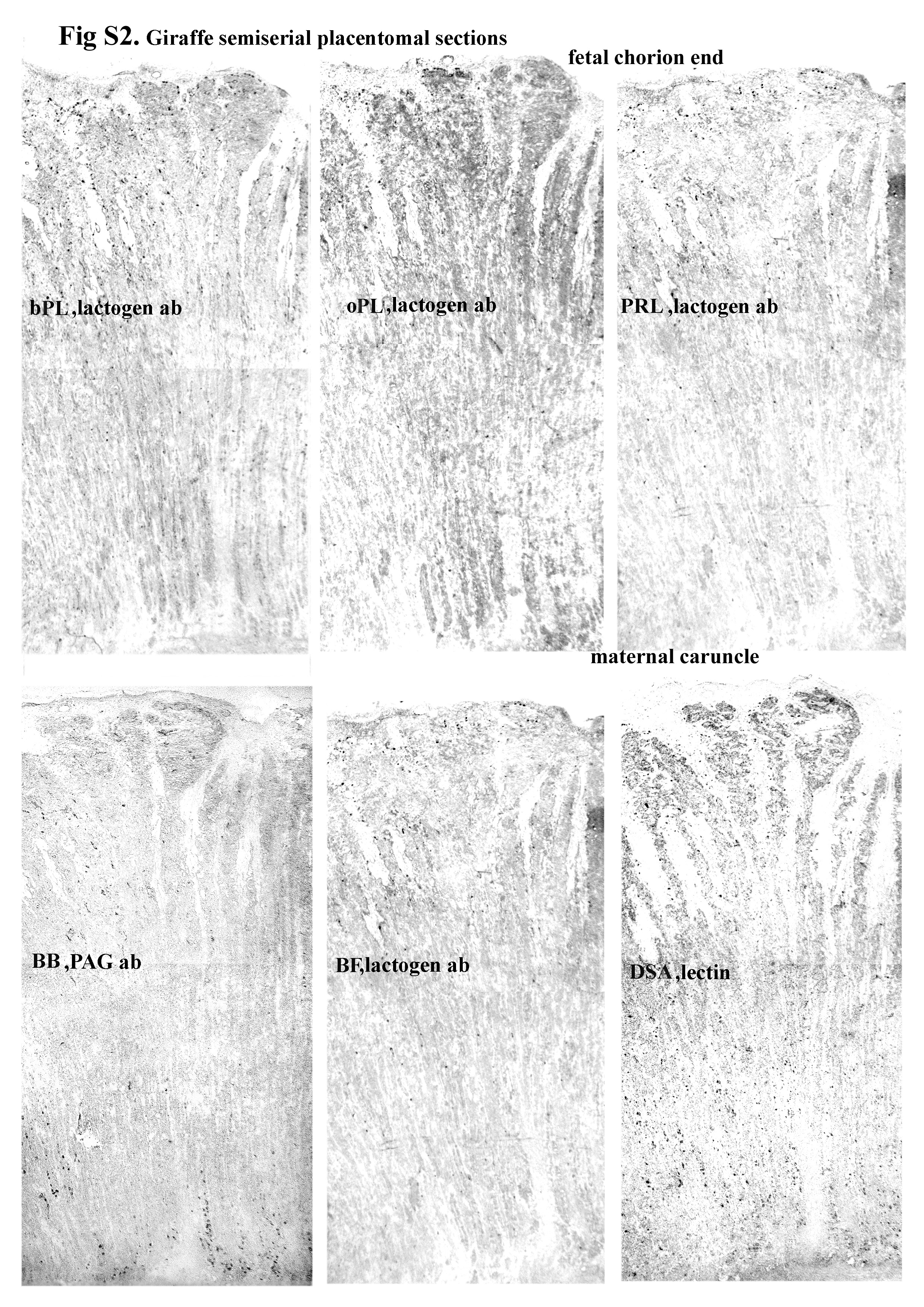

Supplement: FIG_S2_ioab247 [file fig_s2_ioab247.jpeg]

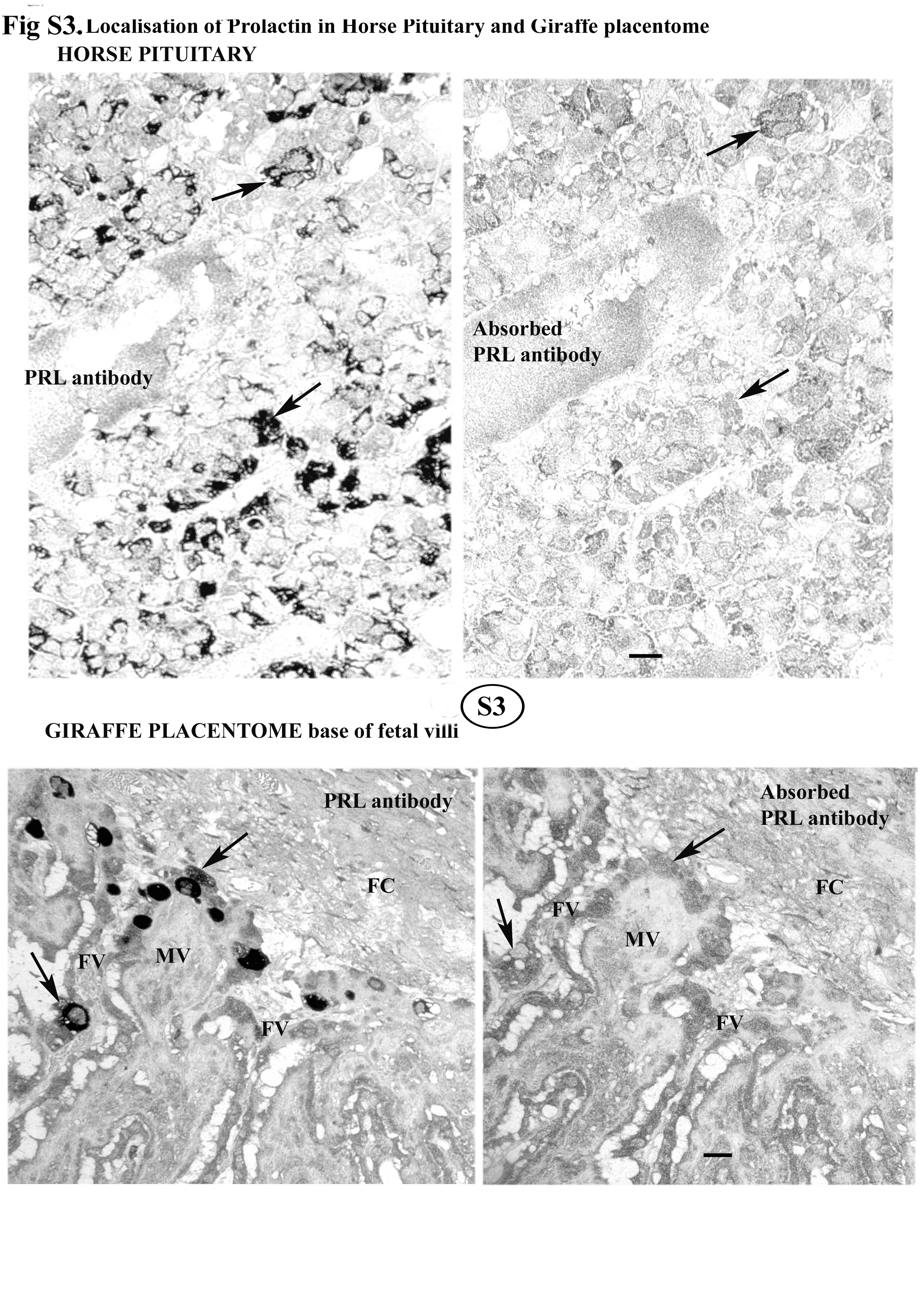

Supplement: FIG_S3_ioab247 [file fig_s3_ioab247.jpeg]
