# Supplementary material for: Asymmetric expression of proteins in the granules of the placentomal Binucleate cells in Giraffa camelopardalis
Source: Biol Reprod. 2022 Jan 17;106(4):814–22. doi: 10.1093/biolre/ioab247 (PMC9305501; doi:10.1093/biolre/ioab247)
Supplement: Table_S1_ioab247 [file table_s1_ioab247.docx]

Table S1. Animal samples: origins.

|  |  |
| --- | --- |
| SPECIES | ORIGIN |
| Cow (Bos taurus)  Ewe (Ovis aries)  Goat (Capra hircus) | Dr F.B.P. Wooding,  Babraham Institute,  Cambridge |
| White-Tail Deer (Odocoileus virginia) | D. Osborn, Warnell School of Forestry and Natural Resources, University of Georgia, USA and Dr G Killian, Pennsylvania State University, USA |
| Fallow deer (Dama dama) and Roe Deer (Capreolus capreolus) | R.Witta, Ranger, Thetford Forest, Norfolk, UK |
| Red deer (Cervus elaphus) | Regents Park, London UK and Dr CL Adam, Rowett Research Institute, Aberdeen,UK |
| Axis Deer (Axis axis);  Chinese Water Deer (Hydropotes inermis);  . | Veterinary Dept, ZSL Whipsnade Zoo, Dunstable, UK. |
| Wildebeest (Connochaetes taurinus) and  Giraffe (Giraffa camelopardalis).  Horse pituitary | Dr WR Allen, The Paul Mellon Laboratory, Newmarket, UK |
| Impala (Aepyceros melampus)  Springbok (Antidorcas marsupialia) | Dr RD Van Aarde, Dr JD Skinner, Mammal Research Institute, University of Pretoria, South Africa. |
| Tragulus (Tragulus spp.) | Dr J Kimura, Seoul National University, South Korea. |
| Pronghorn (Antilocapra americana)  Wapiti (Cervus canadensis)  American Plains Bison (Bison bison) | Dr WJ Silvia, CH Hamilton, Dept of Animal and Food Science, University of Kentucky, USA and Dr TW Geary, US Dept of Agricultural Research Service, Miles City, MT, USA |
| Okapi (Okapia johnstoni) | Dr K Benirschke, Dept Pathology, University of California, San Diego, USA |
